# Nutraceutical Intervention of Probiotics in Vision Science: A Systematic Review of Evolving Perspective

**DOI:** 10.1155/joph/5690407

**Published:** 2026-08-17

**Authors:** Sankhajyoti Saha, Moubani Dutta, Agnihiya Bosu

**Affiliations:** ^1^ Bhawanipur College of Creative Arts and & Applied Sciences, Kolkata, India; ^2^ National University, Gazipur, Bangladesh, nu.edu

**Keywords:** dysbiosis, gastrointestinal microbiome, gut–eye axis, ocular drug delivery, ocular surface microbiota, probiotics

## Abstract

Probiotics are active elements of the gastrointestinal tract that, when administered in adequate quantities, confer prolonged health efficacy on host organisms by enhancing the equilibrium of their gut microbiota (GM). This GM regulates numerous aspects of human wellness and is required for sustaining metabolic equilibrium. A gut–eye axis is a bidirectional mechanism that suggests an interaction between intestinal microbiota and the eyes, which is robust to microbial cultures like ocular surface microbiota (OSM). This review integrated PubMed, Scopus, and Web of Science through a systematic approach and probed the therapeutic efficacy of the human microbiota against the gut–eye homeostasis, which enables the invasion and growth of bacteria in the ocular region, triggering inflammation and deranging regional intestinal immune stability, molecular resemblance, and the subsequent decrease of acceptability concerning ocular antigens. Immunomodulation of the microenvironment and improving the human gut microbiota (HGM) are keys to reducing infections. Probiotics with antibacterial properties, unlike microorganisms that induce eye infections, may significantly reduce infections, making them a potential alternative to antibiotics. The antibacterial activity of probiotic strains significantly reduced bacterial growth, suggesting clinical promise for some chronic neurodegenerative diseases related to commensal bacteria, particularly through immune modulation, nutritional competition, or inhibitory chemical production. Well‐designed clinical experiments in future could explore the antimicrobial spectrum of each strain and how probiotics respond to different pathogens. With optimal delivery methods, an evaluator will likely pave the way for personalized probiotic intervention.

## 1. The Probiotic Profile

The term “Probiotic” is a blend of the Latin word “Pro” and the Greek word “Bio” that means “for life,” and it was first patented by Werner Kollath in 1953, to describe the active elements that stimulate various positive effects, in view of their potential health benefits and by enhancing the equilibrium of its intestinal microbiota, regardless of their form of culinary substances. Food and Agriculture Organization (FAO) and World Health Organization (WHO) designated Probiotics as “live microbes when administered in adequate quantities, confer health benefits on host organisms” [1, 2]. The efficacy of probiotics—identified as microbial culture, comprising live bacterial elements, exhibiting optimal health while administered in substantial quantity—has garnered a lot of concern. The strains of lactic acid bacterium and bifidobacterium are prominent instances that are frequently utilized in efficient nutritional supplements. Trial evidence indicates that they are effective in reducing atopic dermatitis and allergic rhinitis among newborns. Probiotics are additionally being investigated as alternative interventions for metabolic illness, for instance, metabolic syndrome, fatty liver syndrome, and type 2 diabetes, including inflammatory bowel disorder. The aforementioned favorable impacts are facilitated by plenty of strain‐specific systems, spanning immunomodulation, neurotransmitter synthesis, intestinal barrier activity enhancement, and selective eradication of pathogens. Probiotics and pathogens contend for nutrients and receptor‐binding regions by conducting a process known as competitive marginalization, which restricts pathogen colonization. In addition, probiotics contain antimicrobial chemicals that effectively prevent the formation of contaminants, consisting of bacteriocins, hydrogen peroxide, organic acids, and short‐chain fatty acids (SCFAs). By stimulating the integration of mucin and governing coupling proteins like occludin and claudin, the strength of the intestinal lumen is maintained, restricting microbial translocation and minimizing inflammatory responses. Probiotics additionally assist in preserving intestinal homeostasis by monitoring the host’s immunological mechanism. More importantly, the capacity they have to release neurotransmitters promotes an avenue of the gut–brain interactive behavior [1, 3, 4]. By reason of the discernible physiological and anatomical construction, ocular medicament has ventured consequential provocation to pharmacologists and drug delivery professionals. Drug delivery in the eyes is influenced by dynamic (conjunctival and choroidal blood circulation, lymphatic outflow, and tear dynamics), static (distinct segments of precorneal tear film, cornea, sclera, retina that involve the blood–aqueous and blood–retinal interfaces), and efflux pumps (specifically to the posterior segment) impediment. Research has advanced over the past 2 decades to invent safe, patient‐adherent apparatus to deliver medication intensity in ocular tissues [5, 6]. Rapid precorneal medication clearance is one of the main issues, despite having a great acceptance rate. New drug delivery systems for ophthalmic administration are the focus of a lot of work to expand ocular medication bioavailability, which may overcome these obstacles and preserve drug levels in tissues [7]. To get over a heterogeneity of static and dynamic obstacles, an expanding coalition to identify influx transporters on different ocular tissues has been developed to obtain a parent drug delivery system that targets these transporters [5, 8]. The gastrointestinal tract’s microbiota regulates the equilibrium of the digestive system, whereas the OSM inhibits the proliferation of pathogens and is correlated with ocular infections and diseases. Although the human immune system is capable of resisting some bacteria, a sophisticated defensive mechanism precludes microbial growth on the ocular surface. Regardless of having many different antimicrobial defenses, the ocular surface is resilient to microbial cultures like OSM. The tear film is composed of lysozymes, lactoferrin, lipocalin, and β‐defensins, among various anti‐infectious elements. Persistent conjunctival lymphocytes, plasma cells, macrophages, and dendritic cells additionally phagocytose and degrade bacterial connexins [9]. The interaction between gut (the enteric nervous system)–brain (the central nervous system) axis, along with other biological networks, and especially the microbiome of an individual, is currently generating an abundance of concern. The GM regulates numerous aspects of human wellness and is required for sustaining metabolic equilibrium. A gut–eye axis is an idea suggested through published research that evaluates the potential significance of intestinal microbiota restructuring in the occurrence of age‐related macular degeneration, uveitis, glaucoma, and other retinal pathologies. The gut–eye axis, which implies an intriguing link across the pathophysiology of ocular disorders and the gut microbiota, is a manifestation of an extended approach. Considering the gut–eye axis’s mechanics could assist in determining a novel therapeutic regimen for potentially alleviating a spectrum of ocular pathologies [10–12]. Individuals’ gut microbiota compositions are distinctive relative to their diet, lifestyle, and BMI. OSM has emerged as progressively significant due to the notable innovations in 16S rRNA sequencing, exhibiting Proteobacteria as the most prominent, after Actinobacteria, Firmicutes, and Bacteroidetes [9, 13].

Through an extensive review of the availability of evidence on probiotics’ potential advantages for conserving eye health and minimizing the incidence of ocular pathologies, as well as their implied system of action, including immune/inflammatory consequences, gut–eye axis adaptation, and ocular surface microbiome (OSM) impact, this review aims to bridge this knowledge gap in expertise. In addition, it will consider the existing information regarding the efficacy and the reliability of certain probiotic cultivars and substances for an assortment of ocular pathologies. It will also emphasize emerging concerns and advocate prospective study pathways to enhance the potential for therapy of probiotics in eye care.

## 2. Methods

This systematic review adhered to the attentive criterion to synthesize extant documentation on Emerging Applications of Probiotics in Eye care, in accordance with the Preferred Reporting Items for Systematic Reviews and Meta‐Analysis standards.

### 2.1. Literature Source and Search Strategy

Ethical approval was not required since this study involves no direct interaction with human or animal subjects. In order to minimize the risk of reporting bias and establish a transparent inclusion standard, the PROSPERO portal was used to register and publish the study (Registration Number: PROSPERO 2026 CRD420261345593). The literature search was primarily retrieved publications from 2002 to June 2025, besides one seminal study from 1998, with a limit to human studies, and the literature retrieval was completed on March 15, 2026.

The primary search string was formulated in PubMed employing an integration of controlled vocabulary (MeSH terms) and free‐text terms, depicted in Table 1. Subsequently, analogous keyword‐based search strategies were tailored for Scopus and Web of Science. The reference lists of qualifying literature were subjected to a backward snowballing and forward snowballing technique to gather nonindexed literature and databases.

**TABLE 1:** The search strings employed to identify relevant systematic reviews featuring search availability.

Databases	Search string
PubMed	“Dysbiosis”[Title/Abstract] OR “complications”[Title/Abstract] OR “Humans”[Title/Abstract] OR “Prebiotics”[Title/Abstract] OR “Probiotics”[Title/Abstract] OR “Probiotics/therapeutic use”[Title/Abstract] OR “Eye Diseases/immunology”[Title/Abstract] OR “Eye Diseases/metabolism”[Title/Abstract] OR “Eye Diseases/microbiology”[Title/Abstract] OR “Eye Diseases/therapy”[Title/Abstract] OR “Eye/pathology”[Title/Abstract] OR “Gut‐Eye Axis”[Title/Abstract] OR “Humans”[Title/Abstract] OR “Ocular Drug Delivery”[Title/Abstract] OR “Ocular surface microbiota”[Title/Abstract].
Scopus	TITLE‐ABS‐KEY (“Dysbiosis/complications” OR “Gastrointestinal Microbiome/physiology” OR “Humans” OR “Probiotics” OR “Probiotics/therapeutic use” OR “Eye Diseases/immunology”)
AND TITLE‐ABS‐KEY (“Probiotics” OR “Probiotics/therapeutic use” OR “Eye Diseases/immunology” OR “Eye Diseases/metabolism” OR “Eye Diseases/microbiology” OR “Eye Diseases/therapy” OR “Eye/pathology” OR “Gut‐Eye Axis”)	
Web of Science	TS = (“Dysbiosis/complications” OR “Gastrointestinal Microbiome/physiology” OR “Humans” OR “Prebiotics” OR “Probiotics” OR “Probiotics/therapeutic use” OR “Eye Diseases/immunology” OR “Eye Diseases/metabolism” OR “Eye Diseases/microbiology” OR “Eye Diseases/therapy” OR “Eye/pathology” OR “Gut‐Eye Axis” OR “Humans” OR “Ocular Drug Delivery” OR “Ocular surface microbiota”).

*Note:* Source: Researcher’s compilation.

### 2.2. Study Selection and Eligibility Criteria

Studies that described clinical ophthalmic conclusions in English during the predetermined search interval and consisted of peer‐reviewed original human research assessing probiotic interventions in eye care were included. In vitro or animal studies, research that did not examine probiotics as a direct ocular intervention, nonauthored, and studies with insufficient information were also removed. A restricted selection of detected entries has been eliminated all along in the initial assessment, owing to repetition, ensuring that the consolidation is centered on an independent source of information. An apparent limitation is that, regardless of systematic screening initiatives, a limited number of potentially relevant articles were unable to retrieve.

### 2.3. Data Extraction and Quality Assessment

Two independent reviewers (SS and MD) appraised the validity of the study contents employing the Risk Of Bias In Non‐randomized Studies of Interventions (ROBINS‐I) tool across confounding, selection of participants, classification of interventions, deviations from intended interventions, missing data, measurement of outcomes, and selection of the reported result in relation to the review objectives, the adequacy and reproducibility of the search strategy, and the suitability of the information sources and databases searched. To ensure the precision, uniformity, and validity of the extracted data, conflicts and inter‐reviewer disagreements were addressed by discussion and consensus, with arbitration by a third reviewer as needed. In accordance with the ROBINS‐I guidelines, the risk of bias within each domain was subsequently categorized as low, moderate, serious, critical, or “no information.”

### 2.4. Data Synthesized and Risk of Bias

**FIGURE 1:**
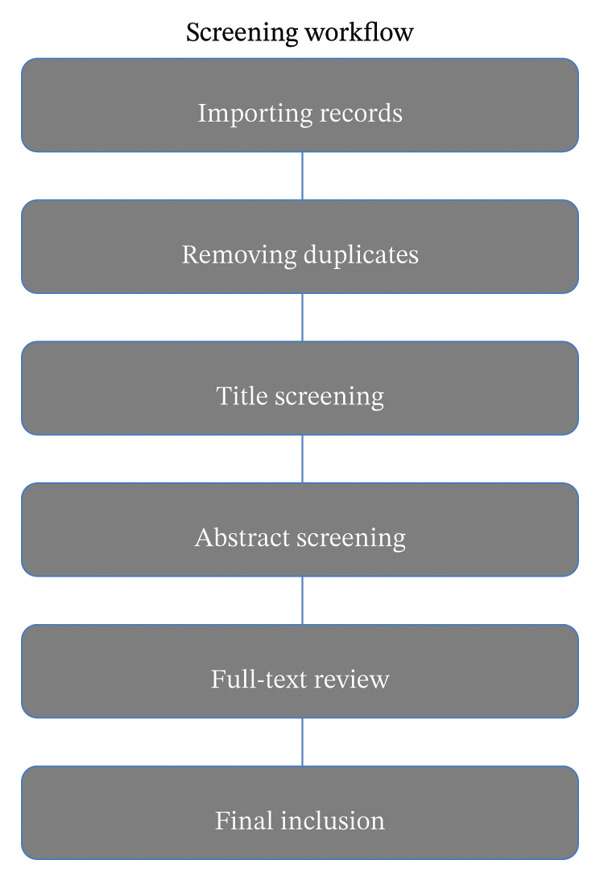
Procedural workflow of the literature screening.

All literature was exported to Microsoft Excel and facilitated the subsequent elimination of duplicates. A preliminary two‐phase screening protocol was implemented: an initial evaluation of titles and abstracts, subsequent to an in‐depth full‐text evaluation, as described in Figure 1. Results were qualitatively synthesized, with extracted studies clustered and screened in accordance with their clinical applications and distinct pathologies. Reviewers systematically assayed each study’s value concerning probiotic medicine and drug delivery—interpreting strains, formulations, routes, mechanisms, and targeted diseases—concurrent with their impact on the OSM, administration routes, and therapeutic role among diverse ocular pathologies.

## 3. Result

### 3.1. Search Results

Prior to formal screening, 57 duplicate articles were removed. In the subsequent retrieval stage, 37 reports were not retrieved. During a comprehensive full‐text review, 89 articles were excluded: among them, 27 contained insufficient information, 29 were wrong interventions, and 33 were restricted to incorrect study design.

**FIGURE 2:**
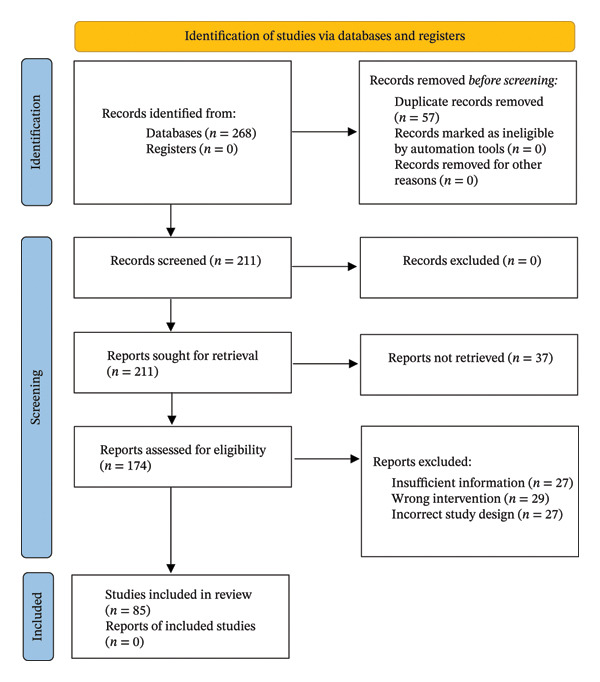
PRISMA flow diagram for literature selection.

This stringent screening process, as illustrated in Figure 1, ensured that the integrated analytical cohort consisted entirely of pertinent clinical evidence. The intention of this detailed and accessible study selection methodology was to circumvent conflict and/or influence and to convey a clinically relevant overview of the information at our disposal, portrayed in Figure 2 [14].

### 3.2. Probiotics in Medicine and Drug Delivery

Nearly 400 strains of bacteria have been determined to inhabit the human gut, and they sustain several kinds of metabolic mechanisms of metabolism that are integral to human health, while contaminants from unsuitable medication usage or defiled substance consumption may disrupt these microbial properties. As a countermeasure to harmful contaminants, probiotics—beneficial bacteria—can contribute to reestablishing consistent functioning that has been disturbed by toxins [15].

Circumstances of the disease, physical endeavors, antibiotic utilization, and nutritional substances are some of the elements that might influence the qualitative and quantitative compositions of the GM. Administration of prebiotics, probiotics, synbiotics, and postbiotics is considered a potential and prospective therapeutic intervention for the cure and/or prevention of several medical conditions, particularly inflammatory, metastatic, cardiovascular, and neurodegenerative illnesses. Preserving the natural gastrointestinal microflora and preserving an optimal gut microbial ecology, probiotics have an extensive spectrum of biological initiatives, including fighting against infections that are resistant to drugs, which is very indispensable for averting more complications. Drug administration techniques that utilize probiotics have additionally prompted consideration since they have inherent positive aspects, consisting of antibacterial execution, host immune response oversight, and the reduction of pathogenic organism infiltration and cellular adsorption, alongside functioning as efficient therapeutic subordinates. They might constitute a secure and cost‐effective choice for administering macromolecules such as enzymes, cytokines, and recombined proteins [15–23]. Table 2 illustrates probiotic strains evaluated, their mechanisms of action, the corresponding study designs, levels of evidence, and supporting references. Relying on the strains, therapeutic regions, and administration procedure, probiotic composition techniques differ.

**TABLE 2:** Modulation of probiotic formulation delivery platform and mechanism.

Probiotic strain	Mechanism	Study type	Evidence level	References
*Enterococcus faecium* CRL183	Anti‐*Candida* biofilm: film inhibited *C. albicans* biofilm (68.9% early, 91.2% mature)	In vitro (film)	Preclinical	[24, 25]
*Lactobacillus brevis* CD2	Anti‐inflammatory (arginine deiminase); buccal film sustained viability (∼99%) and prolonged mucosal contact	Formulation (buccal film)	Preclinical	[26]
*Lactobacillus casei* IFO 15883	Spray‐freeze‐dried powder: retained ∼97.7% viability under optimal conditions	Formulation (spray‐freezing)	Preclinical	[27]
*Lacticaseibacillus rhamnosus* GG	Spray‐freeze‐drying: retained ∼7.4 log CFU/g (≈1‐log loss) viability; ∼92% yield	Formulation (spray‐freezing)	Preclinical	[28]
*Lactobacillus acidophilus* ATCC 4962	Enteric‐coated granules: protected from gastric acid; release at intestinal pH improved survival	Formulation (enteric‐coated)	Preclinical	[29]
*Limosilactobacillus reuteri* (strain)	Anticariogenic: inhibited *Streptococcus mutans/sobrinus* growth and biofilm formation in vitro	In vitro	Preclinical	[30]
*Lactobacillus reuteri* LRT18	pH‐sensitive tablet (phthalyl‐inulin): enhanced gastric survival vs. solution; rapid intestinal release; 6‐mo stability	Formulation (tablet, simulated GI)	Preclinical	[31, 32]
*Lactiplantibacillus plantarum* Lp299v	Orodispersible tablet w/mucoadhesive polymer (Carbopol 971P): strong buccal adhesion.	Formulation (tablet stability)	Preclinical	[33–35]
*L. plantarum* ATCC 13643	Pectin/starch hydrogel encapsulation: greatly enhanced survival in SGF/SIF (encapsulated 5.15–6.67 log CFU/g vs ∼0 for free cells)	Formulation (hydrogel)	Preclinical	[36, 37]
*Streptococcus salivarius* 24SMB	Nasal spray: bacteriocin‐producer; reduced AOM recurrence (30.0% vs 14.9% AOM‐free in treated vs placebo)	Clinical trial (RCT)	Human clinical level	[38]
*S. oralis* 89a + *S. salivarius* 24SMB	Nasal probiotic mix: safe prophylaxis; reduced recurrent AOM in children (observational study)	Observational	Clinical	[39]
*Lactobacillus spp*. (unspecified)	Probiotic eye drops (Lactobacilli): pilot study reported symptom improvement in allergic conjunctivitis (VKC)	Clinical case series	Preclinical	[40]

*Note:* Source: Researcher’s compilation.

### 3.3. The OSM

#### 3.3.1. Probiotic Reliant Ophthalmic Drug Dispensing Framework

Numerous eye issues have been believed to result from dysbiosis, or an imbalance in the GM [41]. By inducing a disruption in the immunological homeostasis of the gut, dysbiosis may trigger inflammation and accelerate faster immune activation processes. The impairment of immune homeostasis, which enables the invasion of bacterial substances and the growth of immune cells in distant regions, the disruption of regional intestinal immune stability, molecular resemblance, and the subsequent decrease of acceptability concerning ocular antigens are some of the speculations that combine the gut microbiome and ocular illness. Predominantly, the blood–ocular barrier conceals ocular antigens, and comprehending the source of these disruptions is fundamental to interpreting its ability to affect the metabolism of cells [13, 42]. Microbial proliferation and inflammation of the eyes may arise from an unstable ocular microbiome. The microbiota of human beings comprises different kinds of bacteria that inhabit the skin, eyes, stomach, and the inside of the mouth. Ocular surface homeostasis is sustained in a significant amount by an OSM macular degeneration, uveitis, diabetic retinopathy, dry eye, and glaucoma may be modulated by the gut–eye axis, according to the current literature.

#### 3.3.2. The Role of Specific Microbial Phyla in Ocular Disease Severity

Emerging evidence indicates that within specific taxonomic phyla—notably *Firmicutes, Bacteroidetes, Actinobacteria, Proteobacteria, Fusobacteria,* and *Verrucomicrobia*—it may be intimately linked with the onset and severity of distinct ophthalmic pathologies [9]. Age‐related macular degeneration, uveitis, diabetic retinopathy, dry eye syndrome, and glaucoma are among the visual disorders in which immunity alters, considering the gut–eye concordance [12, 43]. Increasing bacterial count was observed in glaucoma cases, and human gut microbiota (HGM) was predictive of age‐related macular degeneration. The literature additionally demonstrated effective variation in HGM between healthy individuals and those suffering from fungal keratitis. Aspergillus and Malassezia fungi were more prevalent in fecal samples from patients with bacterial keratitis, whereas infection involving P. aeruginosa was more prevalent in corneal pathology. Serotonin (5‐HT) is a protective substance against retinal damage, and gut spore‐producing bacteria are mainly liable for constraining its production. The initiation and development of AMD characteristics in animal models have been proven by transforming HGM with a low‐glycemic regimen. Evidence implies an interaction between ocular condition and responses to HGM, accentuating the need for a modality to compensate for the combined effects of OSM and HGM [9]. Some chronic neurodegenerative diseases may also be related to commensal bacteria, which may contribute to the onset and/or progression of specific ocular pathologies [44]. The well‐documented immunomodulatory outcomes of probiotics in allergic inflammation, along with the evidence that vernal keratoconjunctivitis (VKC) remains not solely IgE‐involved, as demonstrated by Iovieno et al. [40], topical Lactobacillus acidophilus ophthalmic solution could represent an innovative approach to therapy to prevent the manifestations of VKC in children. After 4 weeks of therapy, the patient reported noticeable improvement in chemosis, conjunctival hyperemia, tearing, and itching. An obtainable downregulation of TLR‐4 and ICAM‐1 has been verified by impression cytology. TLR‐4 stimulation leads to an activation of ICAM‐1, a cell membrane protein that drives macrophage phagocytosis and triggers inflammation [40, 45].

#### 3.3.3. The Gut–Eye Axis

**FIGURE 3:**
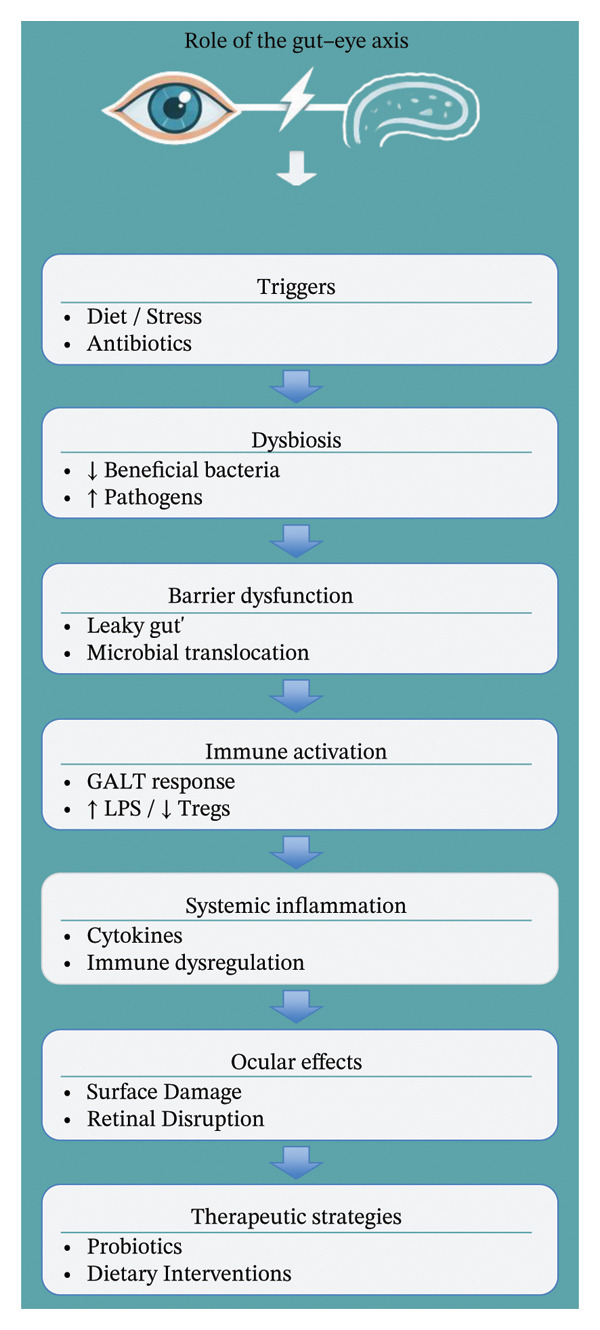
The role of gut–eye axis.

The intricate framework associated with bidirectional interaction involving the GM and the eyes is frequently referred to as the gut–eye axis. Ophthalmic health is modulated by gut health, and conversely, ophthalmic illness tends to affect the gut microbiome, which can potentially have a profound effect on general wellness [9]. Systematically enhancing barrier integrity, balancing systemic immunological and inflammatory pathways, these processes can regulate inflammatory activity, tear film stability, and ocular surface immunity. Figure 2, which describes therapeutic intervention to restore the gut–eye equilibrium and demonstrates the way gut dysbiosis triggers GALT‐mediated immunological and inflammatory pathways, results in systemic transmission and ocular surface and retinal ailments [46–48]. It articulates the advancing comprehension of an analogous link, enabling the equilibrium state of GM, which may govern ophthalmic health and the emergence of eye disorders. Gut dysbiosis, a disparity across the microbial fauna of the gut, might perturb the homeostasis of this summit and contribute to age‐related macular degeneration, uveitis, diabetic retinopathy, dry eye, and glaucoma. Through modulating the immune response, sustaining the blood–retinal barrier, and synthesizing appropriate metabolic substances, gut dysbiosis affects ocular health, as detailed in Figure 3 [9, 41–43].

The reason is its firm structure and potent blood–retinal barrier, which protects cells and molecules from infections. However, environmental stimuli, including allergens and microbes, are constantly present on the outer surface of the eye. OSM is one of the defensive applications that the eye has designed to circumvent sickness [9].

#### 3.3.4. Administration Routes

Probiotics given orally or via transplantation of fecal matter tend to be reliable as novel approaches for ocular infectious conditions. Probiotic eye substances have been established for topical use to treat VKC but not for infectious ailments. The gut–eye axis delivers the framework for the first two; anticipating deviations in HGM may have favorable and/or therapeutic influences upon ocular illness. The eventual method of topical application is an instinctive intention that alters the ocular microenvironment and directly alters OSM [9].

#### 3.3.5. Defiance in Ophthalmic Drug Delivery

Probiotic insertion with ocular administration of medication is an emerging field with intriguing therapeutic potential.

##### 3.3.5.1. Delivery Strategies and Sustainability

The gut–eye axis pathway is fundamental for both oral and fecal avenues, and perturbations in the GM can potentially have an influence on the state of the eye. The indirect feature of this association, yet, makes it difficult to attain reliable outcomes for therapy, emphasizing the evolving significance of OSM. Comprehensive explorations of the OSM, considered significant in conserving homeostasis and modulating the immune system, are being rendered by new sequencing innovations [9]. Gut‐associated lymphoid tissue (GALT) and mucosa‐associated lymphoid tissue (MALT) synchronize immunological control and intestinal homeostasis, establishing the gut–eye axis as the molecular basis for the therapeutic properties of probiotic nutraceuticals in ophthalmic research. GALT structures, comprising Peyer’s patches and isolated lymphoid follicles, sample luminal antigens through M cells, contributing to microbiota‐dependent IgA secretion and Treg/Th17 balance to preserve barrier effectiveness and sensitivity. MALT expands this monitoring to control systemic immunity, where cytokine homeostasis (elevated IL‐17/IFN‐γ) is upset by dysbiosis, facilitating inflammation of the eye in dry eye disease (DED). By reinstating GALT maturation, enhancing sIgA reactions, and inhibiting pathogenic Th1/Th17 skewing, probiotic therapies (Lactobacillus rhamnosus, Bifidobacterium breve) reduce subsequent neuroinflammation that affects corneal nerves and lacrimal glands. By stabilizing MMP‐9/TNF‐α in tears, probiotics’ manipulation of GALT B‐cell niches alleviates DED symptoms, according to the emerging 2024–2025 research that connects gut lymphoid homeostasis to ocular surface repair [49–52].

##### 3.3.5.2. Viability and Stability

Additional exploration into the “antimicrobial spectrum” of each strain and how probiotics respond to pathogens should be done before using them as a novel therapy for complications with the eyes. The effectiveness, clinical results, and affordable output proportions of probiotics, accessible antibiotics, and antiviral drugs should be compared in future studies. Only when a large number of live cells reach the ocular surface can probiotics have a beneficial impact. Defense systems against germs are present on the ocular surface, and probiotic bacteria might not be able to endure over time. Beyond that, the ocular surface is subjected to several variables that may affect the survival of probiotic bacteria, such as individual behaviors, the use of contact lenses, eye rubbing, artificial tears, and systemic ailments. Probiotics have antibacterial effects, while human clinical research frequently demonstrates their integrity and effectiveness. Multidrug‐resistant bacteria, in particular MDR‐PA (multidrug‐resistant Pseudomonas aeruginosa) and MRSA (methicillin‐resistant Staphylococcus aureus), have been suspected for ocular infections as a result of the excessive application of antibiotics. Probiotics have been proven to have antibacterial properties, contrary to microorganisms that induce eye infections. Consequently, microbiome‐targeted approaches may offer potential strategies to address antimicrobial resistance. Preliminary evidence suggests that alterations in the human GM and modulating the microenvironment may influence ocular health through the gut–eye axis, resulting in a potential alternative to antibiotics in future, although this paradigm requires further validation. Probiotics can be taken orally, or by fecal transplantation, and applied topically. To explore their antibacterial activity, a total of six probiotic strains derived from the Lactobacillus and Bifidobacterium groups were established. Notably, each probiotic in this investigation indicated a favorable decline of bacterial proliferation, regardless of antibiotic‐resistant pathogens. However, since OSM experiences variation, the premise of a predominant microbiota is still pragmatic and unpredictable. Ocular toxocariasis might reap benefits from probiotics as an innovative remedy, considering prospective strategies involving immune regulation, nutrition competition, or the synthesis of inhibitory compounds [9].

## 4. Role of Probiotics in Ocular Health

### 4.1. Probiotics for Dry Eye: Mechanism of Action

DED is growing progressively as an intricate inflammatory condition that is modulated by both the immune system and local ocular aspects. Current literature, for instance, a double‐masked, randomized controlled clinical experiment initiated by Tavakoli et al. [53], has established the gut–eye axis’s emerging effectiveness in modulating the ocular surface inflammation, affording an organic rationale for the utilization of probiotics as a remedy for DED. In contrast to a placebo group, a synergistic formulation significantly ameliorated dry eye symptoms, based on a 4‐month clinical investigation executed by Tavakoli et al. The two objective indicators, as noninvasive keratograph break‐up time (NIKBUT) and tear meniscus height (TMH), degraded significantly in the control group while remaining steady in the treatment group, indicating that the substances retained the tear film integrity, disregarding environmental stressors. Immune homeostasis alongside systemic inflammation is substantially maintained among the GM, which is a group of billions of bacteria that reside within the gastrointestinal system. Considering mucosal surfaces are associated with the human organism, encompassing the gastrointestinal and ocular pathways, it has been postulated that dysbiosis occurring in the gut could potentially affect distal mucosal regions, which include the eye, and consequently contribute to the pathology of dry eye. Persons with autoimmune ailments that involve Sjögren’s syndrome, alongside individuals with severe instances of dry eye, have been reported to exhibit impacted gut flora, particularly in shorter diversification. The curative properties of probiotics, which are outlined as viable microorganisms that offer beneficial health effects when delivered in adequate ratios, are accomplished by enhancing mucosal immunological adaptive immunity, minimizing systemic inflammation, and replenishing the GM. The fundamental approach via which probiotics enhance ocular health is immunomodulation [53, 54]. More specifically, probiotics have been observed to modify the extraorbital lacrimal glands’ activity in producing inflammatory cytokines. A course of administration of IRT5 probiotics resulted in a diminution in proinflammatory cytokines containing IL‐1β and IL‐6 and an upsurge in the anti‐inflammatory cytokine IL‐10 in an analogous animal study [55]. These modifications suggest that probiotics could utilize systemic immunological mechanisms to regulate local immune responses at the surface of the eye. In addition, by preventing the degradation of essential tear film components, a probiotic regimen has been established to maintain tear film homeostasis. Probiotics might reduce inflammation, restrict autoreactive T‐cells and cytokines, and potentially stimulate mucin synthesis and epithelial barrier activity, all of which offer the gut–ocular immunological interactions. Extensive research would be helpful to effectively maximize the clinical application while assessing long‐term homeostasis, notably for recipients who are unresponsive to traditional therapies [53, 54].

### 4.2. Meibomian Gland Disease (MGD)

Filippelli et al. [56] suggest that probiotics may improve MGD, specifically chalazion, by influencing the gut–eye axis. The potential approach involves the ability of specific probiotic bacteria to impact the immune system and inhibit inflammation. Several probiotic bacteria, which include Lactobacillus delbrueckii, Streptococcus thermophilus, and Lactococcus lactis, can be beneficial in restricting the biosynthesis of SCFAs, resulting in regulating inflammation, cell death, and immune defenses, specifically in children. The lipid constructions of the meibomian glands can be modified by a disparity in the GM that might culminate in inflammation and the occurrence of chalazion. Probiotics possess the possibility for enhancing the permeability of Meibomian gland secretions by changing the permeability and composition of fat, regulating systemic immunity, and monitoring local cytokine activities, which eventually improve the gut–eye distribution and reclaim a healthy gut biology. The findings of a study suggest that chalazia resolved significantly earlier when pharmaceutical interventions and probiotics were employed concurrently compared to when medical therapy was administered in isolation. Thus, implying that modulating the GM may enhance immune‐mediated stability and minimize inflammation on the ocular surface. Consuming their ability to lessen the inflammation on the ocular surface and enhance tear film stability to relieve contact lens discomfort. The following multifactorial mechanism is noninvasive, safe, and efficient for recurring or persistent chalaziosis, serving as an advantageous adjunct to conventional therapies, potentially reducing the need for surgery [41, 56–58].

### 4.3. Ocular Allergy Management

Probiotics demonstrate clinical efficacy in managing ocular allergic diseases primarily through their immunomodulatory properties and ability to restore GM balance. These beneficial microbes can modulate systemic immune responses by promoting a shift from a Th2‐dominant profile—commonly associated with allergic reactions—to a more balanced Th1/Th2 or regulatory T cell (Treg)–mediated state. They achieve this by enhancing the intestinal barrier, producing anti‐inflammatory cytokines like IL‐10 and TGF‐β, and reducing levels of allergen‐specific IgE. In allergic conditions, including those affecting the eye, such as allergic conjunctivitis, probiotics may mitigate symptoms by decreasing proinflammatory cytokines, inhibiting mast cell activation, and downregulating Th2 and Th17 pathways while upregulating regulatory responses. While much of the current evidence comes from studies on atopic dermatitis, asthma, and rhinitis, the underlying mechanisms suggest potential therapeutic benefits in ocular allergies as well, particularly through systemic immune regulation originating from gut–immune axis interactions [1, 59–62].

### 4.4. Ocular Surface Infection

The OSM, a distinct microbial system, is crucial for immune surveillance and pathogen resistance. A greater susceptibility to infections like conjunctivitis, blepharitis, and microbial keratitis has been connected with disturbance of this sensitive microbiota, which can be provoked by systemic antibiotic consumption, environmental exertion, or chronic diseases. Probiotics prevent by improving both innate and adaptive immune responses, depending on their approach to activity. Beneficial microbe species, such as Corynebacterium mastitidis, stimulate γδ T cells [gamma‐delta (γδ) T cell receptor] to generate interleukin‐17 (IL‐17), which in turn enhances the dissolution of peptides that inhibit microbes into the tear film. Certain peptides, composed of cathelicidins and β‐defensins, promote microbial elimination and maintain the epithelial barrier. Probiotics are additionally helpful in restricting the synthesis of complement components (Proteins) C3 and C9, inflammatory substances consisting of IL‐1β, and secretion of IgA (SIgA), all of which are significant in maintaining the integrity of epithelial cells and resisting microbial invasion. The capability of probiotics to reorient ocular mucosal immunity mediated by microbiome control has been demonstrated by experimental research studies that illustrate topical treatment of beneficial microbes, such as Staphylococcus epidermidis, might rebuild defense against pathogenic organisms like Pseudomonas aeruginosa. Along with the gut–eye axis, probiotics could exert systemic immunomodulatory impacts in contrast to the localized effects on the ocular surface. The proliferation and the formation of biofilm resistant to pharmaceuticals on ocular pathogens, such as Staphylococcus aureus and Neisseria gonorrhoeae, can be prevented by systematic administration of strains such as Lactobacillus rhamnosus and Bifidobacterium spp. in the form of mechanisms like pH imbalance, bacteriocin fabrication, and competitive elimination. Added to that, through controlling receptor manifestation and stimulating regulatory T‐cell exertion, probiotics have been demonstrated in both in vitro and in vivo studies to suppress inflammation‐related processes. As an instance, topical Lactobacillus acidophilus reduces inflammatory responses and TLR4 appearance in VKC. In studies of age‐related macular degeneration and uveitis, probiotic‐persuade components such as tauroursodeoxycholic acid (TUDCA) and SCFAs helped minimizing the oxidative stress and inflammation. Application in the clinical setting remains in stages of development, while strain specificity, colonization propensity on the ocular surface, and efficiency of channel (oral vs. topical) remain obstacles. Notwithstanding such barriers, emerging evidence attests to the immunoprotective advantages of probiotics in managing ocular diseases, indicating the need to conduct additional integrative studies to establish uniform methods of therapy and validate efficiency in experiments on humans [9, 13, 47, 63].

## 5. Aptitude of Probiotic in Ophthalmology

### 5.1. Current Human Clinical Evidence and Limitations

The evidence presented in Table 3 describes the significant methodological constraints, considerably limited cohort sizes (*n* ≈ 7–68), and reduced intervals of observation. The frequent use of multistrain groups and the corresponding administration of standard medications further inhibit the applicability of those outcomes. Methodological consistency is frequently impaired by open‐label designs or the widespread application of a standard‐of‐care approach among all experimental groups (e.g., as observed in the chalazion study). In addition, the selection of placebo components may inevitably introduce confounding variables by independently modifying gastrointestinal or immunological parameters.

**TABLE 3:** Summary of clinical studies evaluating probiotic interventions.

Study population (*n*)	Intervention	Intervention period	Clinical endpoints	Principal findings	Ref
*n* = 7 (Pediatric cohort; VKC)	*L. acidophilus* Eye drops (2 × 10^8^ CFU/mL, 4 times per day)	4 weeks (1 month)	VKC signs and symptoms; ICAM‐1/TLR‐4 expression	Improvement in conjunctival inflammation and symptoms [1]; pilot (open‐label).	[40]
*n* = 26 (Pediatric cohort; Chalazion)	Oral mix: *S. thermophilus* ST10 + *L. lactis* LLC02 + *L. delbrueckii* (≥ 10^9^ CFU each day) + standard care	Up to 6 months (Evaluated once every 2 weeks up to 6 months)	Chalazion size/resolution time	Faster lesion resolution (shorter time) with probiotics [3]; safe.	[57]
*n* = 20 (Adult cohort; chalazion)	Oral mix: *S. thermophilus* ST10, *L. lactis* LLC02, *L. delbrueckii* (DSMs as above) + standard care	Up to 3 months	Chalazion resolution time	Small chalazia resolved quicker on probiotics (21.0 ± 7.0 vs 38.5 ± 9.0 days, *p* = 0.039) [4]; no effect on larger lesions.	[56]
*n* = 41 (Adult cohort; Dry eye)	Oral multistrain probiotics + prebiotics (formulation not detailed)	4 months	OSDI, DEQ5, tear osmolarity, NIKBUT, Schirmer, staining, TMH, lipid layer, redness	Significantly reduction in OSDI than placebo (16.8 vs 23.4, *p* < 0.001); Tear stability maintained against decreases in controls.	[53]
*n* = 41 (Adult cohort; Dry eye)	Oral multistrain probiotics + prebiotics (formulation not detailed)	4 months	Tear MMP‐9, TIMP‐1; serum CRP	No significant alterations in inflammatory biomarkers against placebo.	[63]
*n* = 68 (Adult cohort; Dry eye)	Oral *Streptococcus thermophilus* iHA318 (probiotic)	35 days (Around 5 weeks)	Tear volume, TBUT, osmolarity, serum sialic acid, OSDI, ocular NLRP3	Probiotic group possessed elevated tear volume, prolonged TBUT, reduced osmolarity, enhanced OSDI and decreased NLRP3 signal; substantial symptom relief against placebo.	[13]
*n* = 40 (Adult cohort; Dry eye)	*L. sakei* (lysate) eye drops vs placebo; *L. sakei* oral capsules vs placebo (2 × 2 design)	Not specified	OSDI, TBUT, Schirmer I, tear IL‐6/TNFα/IFNγ	Topical L. sakei drops significantly enhanced OSDI, TBUT, Schirmer (*p* < 0.0001) and reduced tear cytokines. Oral L. sakei had no added benefit.	[41, 47, 64, 65]

### 5.2. Variability and Methodological Limitations in Probiotic Research

Since probiotic benefits are strain‐specific, it is difficult to generalize findings to other strains of bacteria. The existence and efficacy of closely associated strains are influenced by considerable variances in factors like oxygen or acid resistance. For instance, freeze‐dried Lactobacillus rhamnosus GG decreases its efficacy by 30%–40% regardless of whether it has good acid tolerance. The difficulties in comparing studies that use diverse formulations—comprising different species, strains, dosages, and delivery methods—because of their strain‐specific character have been noted. Moreover, the lack of a defined approach results in disagreements over product characteristics, dose, and strain preference, making it more difficult to assess probiotic health claims [66, 67]. The generalization of probiotic outcomes from animals to humans is another significant concern. Animal or in vitro research offers an extensive amount of mechanistic and efficacy data. Considering that much research primarily uses animal models, Latif et al. point out the need to extend these findings to the human context. Animal experiments can provide insight into mechanisms that include immune regulation across the gut–eye axis, but physiological variations, differences in the microbiome, and distinct disease factors may limit the results’ significance to humans. Consequently, in order to establish their potential benefits for patients, any prospective probiotic effects identified in animal or cell culture experiments, well‐planned human trials are mandatory for validation [48, 67].

Probiotics used orally or by fecal transplantation are generally dependable as innovative treatments for infectious diseases of the eyes. Probiotics for the eyes have been developed for the topical treatment of inflammation but not for infectious diseases. Regardless of antibiotic‐resistant pathogens, the antibacterial activity of six probiotic strains of Lactobacillus and Bifidobacterium was investigated. The results showed a good drop in bacterial growth. A novel treatment for ocular toxocariasis, probiotics considering immune modulation, nutrition competition, or inhibitory chemical manufacturing techniques are guiding us with clinical promises for some chronic neurodegenerative diseases that are related to commensal bacteria.

### 5.3. Future Prospects

Though probiotic therapies enhance certain ophthalmic outcomes, precisely dry eye illness, pursuant to existing evidence, some of the enhancements are extremely strain‐, formulation‐, and condition‐specific instead of a general “ocular benefit” of probiotics. In dry eye cases, specific strains such as Lactobacillus plantarum NK151, Bifidobacterium bifidum NK175, and mixtures such as IRT5 improve tear production, maintain goblet cells, reduce corneal staining, and reduce ocular and lacrimal inflammatory markers while modifying the gut microbiome and raising IL‐10:TNF‐α ratios in colon and ocular tissues [68–71]. Comparable advantages were identified with Streptococcus thermophilus iHA318, which reduces oxidative stress and inflammatory cytokines while retaining tear film integrity in UVB‐associated dry eyes [72]. Human information, instead, exhibits greater complexity. Oral symbiotics enhanced OSDI grades and sustained noninvasive tear break‐up time (TBUT) and tear meniscus height compared to a reduction observed in placebo groups, [53, 72, 73]^.^ Oral S. thermophilus iHA318 enhanced tear volume, TBUT, decreased osmolarity, and inhibited NLRP3 activation in a randomized experiment [13]. A factorial RCT on dry eye revealed that a Latilactobacillus sakei lysate eye drop (postbiotic) considerably improved symptoms, signs, and tear cytokines, even if the associated oral probiotic capsule possessed no discernible impact [47]. Another randomized controlled trial revealed no alteration in systemic or tear inflammatory indicators following probiotic supplementation, albeit perceived clinical enhancement, with inflammatory markers increasing postcessation [63]. Considering the gut–eye axis, consistent emphasis on sample sizes, brief follow‐up periods, heterogeneous outcome metrics, and frequently absent mechanism‐centered methods, it is concluded that microbiome‐derived nutraceuticals in ophthalmology deserve to be considered purely experimental, strain‐specific adjuncts instead of generalized efficacious ocular interventions [9, 12, 74–78].

## Funding

No funding was received for this manuscript.

## Conflicts of Interest

The authors declare no conflicts of interest.

## Data Availability

The data that support the findings of this study are available from the corresponding author upon reasonable request.
